# Nomogram to predict hemorrhage risk related to anti-tumor therapy in patients with acute leukemia

**DOI:** 10.3389/fonc.2025.1684145

**Published:** 2026-02-26

**Authors:** Xinxin Hu, Ying He, Yinghua Xie, Li Zhao, Ruijuan Wang, Lijuan Duan, Peipei Mao, Xiyao Han, Yihan Liu, Chao Li

**Affiliations:** 1 Department of Hematology, Shanghai Fifth People’s Hospital, Fudan University, Shanghai, China; 2 Department of Hematology, Nanyang Municipal Central Hospital, Nanyang, Henan, China; 3 Clinical Molecular Cytogenetics and Immunology Laboratory, The First Hospital of Lanzhou University, Lanzhou, Gansu, China

**Keywords:** hemorrhage, nomogram, acute leukemia, anti-tumor therapy, predictive model

## Abstract

**Introduction:**

This study aimed to identify clinical characteristics associated with hemorrhagic events in acute leukemia patients who received anti-tumor therapies and to develop and evaluate a prediction nomogram for hemorrhagic events based on those characteristics.

**Method:**

This retrospective cohort study included 468 acute leukemia patients, excluding those with acute promyelocytic leukemia, treated at The Shanghai Fifth People’s Hospital and Nanyang Municipal Central Hospital between January 2013 and December 2023. The primary endpoint was World Health Organization (WHO) grade 2 or higher hemorrhagic events related to anti-tumor therapy. Patients were randomly divided into training and test groups at a ratio of 7:3. In the training group, univariable logistic analysis and least absolute shrinkage and selection operator (LASSO) regression were performed to identify significant predictors, which were then used to construct a prediction nomogram for hemorrhage risk. Nomogram performance was evaluated by receiver operating characteristic (ROC) curve analysis, calibration curve analysis, and decision curve analysis (DCA). The following five independent variables were identified as predictors of anti-tumor therapy-related hemorrhagic events in acute leukemia patients and used to develop a prediction nomogram: infection status, types of different hemorrhage prevention drugs and blood products administered, platelet (PLT) transfusion, hematocrit, and PLT count.

**Result:**

On ROC curve analysis, the nomogram exhibited satisfactory performance in both the training group [area under the ROC curve (AUC)=0.741] and test group (AUC=0.718). Calibration plots showed a high degree of consistency between the actual and nomogram-predicted survival rates in both groups, and the nomogram showed good clinical utility on DCA. We successfully developed and validated a nomogram for predicting the risk of anti-tumor therapy-related hemorrhage of WHO grade 2 or higher among patients with acute leukemia.

**Conclusion:**

This nomogram may provide a practical and user-friendly tool for clinical practice once further validated in perspective large cohort or trials.

## Introduction

Acute leukemia is a rapidly progressing hematologic malignancy caused by clonal proliferation of hematopoietic stem cells (1). According to the major cell types involved (2), it is classified as acute myeloid leukemia (AML) or acute lymphoblastic leukemia (ALL). Its characteristics include rapid progression, short duration, and poor prognosis (3, 4). According to World Health Organization (WHO) statistics, 474,500 cases of leukemia were newly diagnosed worldwide in 2020, accounting for 2.46% of all new cancer cases globally (5), while in China specifically, 85,400 cases were newly diagnosed in 2020, accounting for 1.87% of all new cancer cases. Leukemia is among the top 10 malignant tumors causing death in Chinese residents and has the highest incidence and mortality of malignant tumors among children and individuals less than 35 years of age (6).

Hemorrhage, a common complication in patients with leukemia after treatment with chemotherapy or targeted therapies, is caused by a variety of factors such as thrombocytopenia, reduced levels of coagulation factors, platelet (PLT) dysfunction, and vascular damage (7). It is also a major cause of mortality for leukemia patients (8, 9). To reduce the risk of hemorrhage, prophylactic PLT transfusions guided by the patient’s PLT count are typically administered in clinical practice (10, 11). However, hemorrhagic events still occur frequently. According to Stanworth et al., among chemotherapy-treated patients with malignant hematological tumors who received PLT transfusions, the incidence of WHO grade 2, 3, or 4 hemorrhagic events is as high as 43% (12). Accordingly, the identification of risk factors for hemorrhagic events related to anti-tumor therapy is of great importance for improving therapy outcomes and patient prognosis.

Previous studies have explored factors related to hemorrhagic events after anti-tumor therapy. A study by Masternak et al. found that mean PLT volume (MPV), a readily available hematological marker, is closely associated with prognosis after chemotherapy and the incidence of adverse events in patients with hematologic malignancies (13). In another study, lactate dehydrogenase (LDH)/fibrinogen (FBG) levels and FBG response were found to be linked to morbidity and mortality from hemorrhage in patients with acute promyelocytic leukemia (14). However, an effective risk prediction model for hemorrhagic events in these patients has yet to be developed.

To fill this research gap, the present study aimed to identify risk factors for WHO grade 2 or higher hemorrhagic events among acute leukemia patients after anti-tumor therapies and to develop a risk prediction model for these events. The results of this study provide insight for early identification of high-risk patients, which will support the prevention of hemorrhagic events and improve patient outcomes.

## Methods

### Study design and data sources

This was a retrospective cohort study, and the data were obtained from Shanghai Fifth People’s Hospital and Nanyang Municipal Central Hospital for cases treated between January 2013 and December 2023. The research protocol was approved by the Ethical Committee of Shanghai Fifth People’s Hospital, Fudan University (2023, ethical approval record No. 076). The study was also conducted in accordance with the tenets of the Declaration of Helsinki.

### Study eligibility criteria

The inclusion criteria were as follows: (1) age ≥18 years; (2) new diagnosis of acute leukemia; and (3) receiving initial treatment with chemotherapy, targeted therapy, or a combination of chemotherapy and targeted therapy. The exclusion criteria were as follows: (1) diagnosis of acute promyelocytic leukemia (n=133); (2) hemorrhagic events of WHO grade 2 or higher within 2 weeks prior to therapy (n=110); (3) history of congenital or acquired coagulation disorders (n=2); (4) primary malignant tumors in other systems (n=3); (5) severe functional impairment of vital organs such as heart, liver, and kidneys (n=8); (6) pregnancy or lactation (n=3); and (7) incomplete clinical data (n=2). A total of 468 participants were included in the final study. A flow chart outlining patient selection is presented in **Supplementary Figure 1**.

### Data collection

#### Outcome variables

The primary endpoint was hemorrhage related to anti-tumor therapy with a severity of WHO grade 2 or higher, which was defined as any hemorrhagic event that occurred after the start of an anti-tumor therapy and before recovery (defined as PLT count >75×10^9^/L and absolute neutrophil count [ANC] >1.5×10^9^/L) from myelosuppression during anti-tumor therapy in patients. The WHO grading system is widely utilized to assess the severity of hemorrhagic events in PLT transfusion trials, which classifies hemorrhagic events into the following grades: grade 1 (mild), grade 2 (moderate; immediate red blood cell [RBC] transfusion not required), grade 3 (severe; necessitating RBC transfusion within 24 hours), or grade 4 (debilitating or life-threatening) (15–17). The secondary outcomes were overall survival (OS) and event-free survival (EFS). EFS was defined as the number of days that elapsed from the beginning of treatment until disease progression, recurrence, or all-cause mortality. Recurrence was defined as the reappearance of at least 5% of bone marrow blast cells or abnormal cells after a patient achieved complete remission and/or extramedullary infiltration of leukemia cells. Disease progression was defined by a 25% increase in the absolute number of primitive cells in the peripheral blood or bone marrow, or the appearance of extramedullary disease.

#### Follow-up

Follow-up was conducted at 3-month intervals during the first 1 year after completion of treatments and at 6-month intervals thereafter. The relevant diagnosis and treatment information were queried from the electronic medical record management system of each inpatient or outpatient clinic. If patients were lost to follow-up during the study period, the most recently recorded data were utilized. Information regarding mortality was obtained through meticulous examination of hospital records and death certificates, or via direct communication with the patient’s relatives or referring physicians. Patients were followed up until disease progression, recurrence, death, or the end point of follow-up, which was December 31, 2023.

#### Other variables

Additional clinical information was collected for the study participants. The following data were obtained from before the start of anti-tumor therapy: (1) demographic information: age, gender, body mass index (BMI, kg/m^2^), smoking history, drinking history, family history of leukemia, family history of solid cancer, history of blood disorders (such as myelodysplastic syndrome, lymphoma, polycythemia vera, multiple myeloma, hemophilia, disseminated intravascular coagulation [DIC], splenomegaly, etc.), and comorbidities (hypertension, diabetes, coronary heart disease, cerebrovascular disease, and autoimmune disease); (2) disease features: anemia, extramedullary infiltration, hemorrhage, and infection; (3) morphological examination of bone marrow cells; and (4) blood test results: white blood cell (WBC) count (10^9^/L), absolute neutrophil count (ANC) (10^9^/L), lymphocyte (LYM) count (10^9^/L), RBC count (10^12^/L), hemoglobin (Hb, g/L), hematocrit (HCT, %), PLT count (10^9^/L), total bilirubin (TBIL, μmol/L), alanine aminotransferase (ALT, U/L), aspartate aminotransferase (AST, U/L), gamma-glutamyl transferase (GGT, U/L), alkaline phosphatase (ALP, U/L), blood urea nitrogen (BUN, mmol/L), creatinine (Cr, μmol/L), mean platelet volume (MPV, fL), platelet distribution width (PDW, fL), platelet–large cell ratio (P-LCR), prothrombin time (PT, s), activated partial thromboplastin time (APTT, s), thrombin time (TT, s), fibrinogen (Fg), D-dimer (D-D), and uric acid (UA). The following data were obtained during the period of therapy: therapy method (including chemotherapy, targeted therapy, and a combination of both chemotherapy and targeted therapy), types of hemorrhage prevention drugs and blood products, PLT transfusion, and number of treatment sessions. The following data were obtained after the completion treatment: WBC count, ANC count, LYM count, RBC count, Hb, HCT, PLT count, TBIL, ALT, AST, GGT, ALP, BUN, Cr, MPV, PDW, P-LCR, PT, APTT, TT, Fg, D-D, and UA.

### Statistical analysis

Values were recorded for variables with less than 20% missing data, whereas variables with more than 20% missing data were excluded. Sensitivity analyses were conducted to compare the data before and after imputation, and the results are presented in **Supplementary Tables 1, 2**. To assess the distribution normality of continuous variables, the skewness and kurtosis methods were employed. The Levene test was used to evaluate the homogeneity of variance. For normally distributed continuous variables, data were presented as mean±standard deviation (SD), and intergroup comparisons were made using the t-test for homogeneous variances and the t’-test for heterogeneous variances. For non-normally distributed continuous variables, median and interquartile range [M (Q1, Q3)] values were calculated, and intergroup comparisons were made using the Wilcoxon rank sum test. Categorical variables were presented as frequencies and percentages. Fisher’s exact test or Chi-square test was used for comparisons of categorical variables. The multiple interpolation method was applied for variables with a missing proportion ≤ 20%. **Supplementary Table 2** shows the results of sensitivity analyses before and after interpolation.

All included patients were randomly divided into a training group and a test group at a ratio of 7:3, and differences in the distributions of basic characteristics between the training and test groups were analyzed. In the training group, all variables were subjected to univariable logistic analysis to assess their association with the occurrence of hemorrhagic events. Variables associated with hemorrhage with a *P*<0.05 were included in the least absolute shrinkage and selection operator (LASSO) regression analysis to identify potential predictors. Then, a nomogram for predicting the risk of anti-tumor therapy-related hemorrhage of WHO grade 2 or higher was established based on the identified predictors. Receiver operating characteristic (ROC) curve analysis and calibration curve analysis were conducted to evaluate the accuracy and reliability of the developed nomogram in both the training group and test group. Decision curve analysis (DCA) was utilized to validate the clinical applicability of the predictive nomogram. Kaplan–Meier curve analysis and the log-rank test were used to compare survival outcomes (OS and EFS) between patients who experienced WHO grade 2 or higher hemorrhagic events and those who did not. Odds ratio (OR) and 95% confidence interval (CI) values were calculated. Values of *P*<0.05 indicated statistical significance. All statistical analyses were conducted using R software.

## Results

### Patient characteristics

A cohort of 468 patients diagnosed with acute leukemia was recruited for the present study. Subsequently, these patients were divided into two groups: the training group (n=328) and the test group (n=140). Statistical analysis revealed no significant differences in patient characteristics between the training and test groups, indicating a well-balanced distribution of data within the two groups (**Table 1**).

**Table 1:** Comparison of clinical characteristics between the training and test groups.

Variables	Total (N=468)	Training set (n=328)	Test set (n=140)	Statistics	*P*
Demographic data					
Age, years, mean±SD	53.83±14.49	52.58±14.86	53.42±13.62	t=-0.577	0.564
Sex, n (%)				χ²=1.233	0.267
Male	249 (53.21)	180 (54.88)	69 (49.29)		
Female	219 (46.79)	148 (45.12)	71 (50.71)		
Height, cm, mean±SD	165.46±7.96	165.69±7.85	164.91±8.22	t=0.967	0.334
Weight, kg, mean±SD	65.25±11.54	65.49±11.61	64.67±11.38	t=0.701	0.484
BMI, kg/m^2^, mean±SD	23.75±3.44	23.79±3.53	23.68±3.21	t=0.311	0.756
History of cancer, n (%)				χ²=0.034	0.853
No	454 (97.01)	318 (96.95)	136 (97.14)		
Yes	14 (2.99)	10 (3.05)	4 (2.86)		
History of hematological disease, n (%)				χ²=2.982	0.084
No	453 (96.79)	321 (97.87)	132 (94.29)		
Yes	15 (3.21)	7 (2.13)	8 (5.71)		
Smoking, n (%)				–	0.804
Never	377 (80.56)	266 (81.10)	111 (79.29)		
Former	12 (2.56)	9 (2.74)	3 (2.14)		
Current	79 (16.88)	53 (16.16)	26 (18.57)		
Drinking, n (%)				–	0.316
Never	424 (90.6)	299 (91.16)	125 (89.29)		
Former	5 (1.07)	2 (0.61)	3 (2.14)		
Current	39 (8.33)	27 (8.23)	12 (8.57)		
Hypertension, n (%)				χ²=0.136	0.712
No	389 (83.12)	274 (83.54)	115 (82.14)		
Yes	79 (16.88)	54 (16.46)	25 (17.86)		
Diabetes, n (%)				χ²=0.055	0.815
No	430 (91.88)	302 (92.07)	128 (91.43)		
Yes	38 (8.12)	26 (7.93)	12 (8.57)		
Coronary heart disease, n (%)				χ²=0.291	0.589
No	444 (94.87)	310 (94.51)	134 (95.71)		
Yes	24 (5.13)	18 (5.49)	6 (4.29)		
Cerebrovascular disease, n (%)				χ²=0.852	0.356
No	437 (93.38)	304 (92.68)	133 (95.00)		
Yes	31 (6.62)	24 (7.32)	7 (5.00)		
Autoimmune disease, n (%)				χ²=0.02	0.888
No	459 (98.08)	322 (98.17)	137 (97.86)		
Yes	9 (1.92)	6 (1.83)	3 (2.14)		
Comorbidities, n (%)				χ²=1.171	0.279
No	337 (72.01)	241 (73.48)	96 (68.57)		
Yes	131 (27.99)	87 (26.52)	44 (31.43)		
Disease features					
Anemia, n (%)				χ²=0.171	0.679
No	58 (12.39)	42 (12.80)	16 (11.43)		
Yes	410 (87.61)	286 (87.20)	124 (88.57)		
Extramedullary infiltration, n (%)				χ²=0.121	0.727
No	431 (92.09)	303 (92.38)	128 (91.43)		
Yes	37 (7.91)	25 (7.62)	12 (8.57)		
Prior bleeding, n (%)				χ²=2.913	0.088
No	352 (75.21)	254 (77.44)	98 (70.00)		
Yes	116 (24.79)	74 (22.56)	42 (30.00)		
Infection, n (%)				χ²=1.781	0.182
No	212 (45.3)	142 (43.29)	70 (50.00)		
Yes	256 (54.7)	186 (56.71)	70 (50.00)		
Bone marrow cytomorphology					
Acute leukemia, n (%)				–	0.65
ALL	98 (20.94)	68 (20.73)	30 (21.43)		
AML	346 (73.93)	241 (73.48)	105 (75.00)		
Others	24 (5.13)	19 (5.79)	5 (3.57)		
Treatment information					
Treatment modality, n (%)				–	0.135
Chemotherapy	372 (79.49)	61 (18.60)	27 (19.29)		
Targeted therapy	8 (1.71)	258 (78.66)	113 (80.71)		
Both	88 (18.8)	9 (2.74)	0 (0.00)		
Types of hemorrhage prevention drugs and blood products, n (%)				–	0.781
No	38 (8.12)	26 (7.93)	13 (9.29)		
1	370 (79.06)	262 (79.88)	108 (77.14)		
≥2	60 (12.82)	40 (12.20)	19 (13.57)		
Platelet transfusion, n (%)				χ²=0.177	0.674
No	46 (9.83)	31 (9.45)	15 (10.71)		
Yes	422 (90.17)	297 (90.55)	125 (89.29)		
Treatment courses, times, M (Q_1_, Q_3_)	1.00 (1.00, 3.00)	1.00 (1.00, 3.25)	1.00 (1.00, 3.00)	Z=-0.603	0.547
Laboratory tests (post-treatment)					
WBC count, 10^9^/L, M (Q_1_, Q_3_)	1.96 (0.69, 5.21)	1.98 (0.71, 5.01)	1.90 (0.63, 5.75)	Z=-0.087	0.931
NEUT count, 10^9^/L, M (Q_1_, Q_3_)	0.62 (0.14, 2.35)	0.58 (0.12, 2.46)	0.68 (0.17, 2.09)	Z=-0.082	0.935
LYM count, 10^9^/L, M (Q_1_, Q_3_)	0.72 (0.39, 1.39)	0.72 (0.41, 1.37)	0.78 (0.36, 1.45)	Z=-0.090	0.929
RBC count, 10^12^/L, mean±SD	2.42±0.67	2.44±0.69	2.38±0.62	t=0.975	0.33
Hb, g/L, mean±SD	74.55±20.24	75.10±21.04	73.27±18.22	t’=0.947	0.345
HCT, %, mean±SD	22.89±6.32	23.00±6.53	22.64±5.81	t=0.555	0.579
PLT count, 10^9^/L, M (Q_1_, Q_3_)	26.00 (11.00, 66.25)	27.00 (11.00, 70.00)	25.00 (10.00, 56.50)	Z=-0.845	0.398
Fg, g/L, mean±SD	3.74±2.04	3.80±2.14	3.61±1.78	t=0.923	0.356
PT, s, mean±SD	13.27±4.18	13.26±4.32	13.30±3.84	t=-0.097	0.923
APTT, s, mean±SD	29.70±6.44	29.72±6.36	29.63±6.67	t=0.144	0.886
TT, s, mean±SD	15.15±3.34	15.14±3.50	15.19±2.94	t=-0.137	0.891
TBIL, μmol/L, M (Q_1_, Q_3_)	9.30 (6.90, 13.60)	9.30 (6.90, 13.72)	9.40 (6.90, 13.33)	Z=-0.230	0.818
ALT, U/L, M (Q_1_, Q_3_)	16.50 (11.00, 29.00)	17.00 (11.00, 29.00)	15.50 (11.00, 25.25)	Z=-0.798	0.425
AST, U/L, M (Q_1_, Q_3_)	17.00 (12.00, 27.00)	17.00 (12.00, 27.00)	16.00 (12.00, 24.00)	Z=-0.903	0.366
GGT, U/L, M (Q_1_, Q_3_)	34.00 (20.00, 62.25)	35.00 (21.00, 66.25)	31.00 (19.00, 56.25)	Z=-1.507	0.132
ALP, U/L, M (Q_1_, Q_3_)	71.00 (56.10, 97.08)	71.35 (55.68, 100.15)	69.50 (58.08, 88.78)	Z=-0.371	0.711
BUN, mmol/L, M (Q_1_, Q_3_)	5.08 (3.73, 6.26)	5.13 (3.79, 6.29)	4.88 (3.64, 6.21)	Z=-0.867	0.386
Cr, μmol/L, M (Q_1_, Q_3_)	60.66 (50.00, 74.22)	60.85 (50.98, 75.01)	60.41 (48.63, 72.74)	Z=-1.145	0.252
Outcome					
Bleeding grade, n (%)				–	0.998
No	130 (27.78)	91 (27.74)	39 (27.86)		
Grade 1	54 (11.54)	38 (11.59)	16 (11.43)		
Grade 2	233 (49.79)	164 (50.00)	69 (49.29)		
Grade 3	41 (8.76)	28 (8.54)	13 (9.29)		
Grade 4	10 (2.14)	7 (2.13)	3 (2.14)		
WHO grade 2 or higher bleeding, n (%)				χ²=0	0.993
No	184 (39.32)	129 (39.33)	55 (39.29)		
Yes	284 (60.68)	199 (60.67)	85 (60.71)		
Treatment efficacy, n (%)				–	0.337
Complete remission	165 (35.26)	121 (36.89)	44 (31.43)		
Complete remission with incomplete hematologic Recovery	6 (1.28)	5 (1.52)	1 (0.71)		
Partial remission	15 (3.21)	11 (3.35)	4 (2.86)		
No remission	148 (31.62)	94 (28.66)	54 (38.57)		
Bone marrow not assessed	134 (28.63)	97 (29.57)	37 (26.43)		
Mortality, n (%)				χ²=0.681	0.409
No	363 (77.56)	251 (76.52)	112 (80.00)		
Yes	105 (22.44)	77 (23.48)	28 (20.00)		
Recurrence, n (%)				χ²=0.355	0.551
No	315 (67.31)	218 (66.46)	97 (69.29)		
Yes	153 (32.69)	110 (33.54)	43 (30.71)		
Progression, n (%)				χ²=0.166	0.683
No	454 (97.01)	317 (96.65)	137 (97.86)		
Yes	14 (2.99)	11 (3.35)	3 (2.14)		
EFS, days, M (Q_1_, Q_3_)	144.50 (39.25, 346.00)	152.50 (39.00, 364.25)	110.50 (41.00, 280.25)	Z=-1.244	0.214
OS, days, M (Q_1_, Q_3_)	179.00 (41.00, 404.75)	196.50 (39.75, 432.25)	131.50 (41.00, 348.50)	Z=-1.333	0.183

SD, standard deviation; M, median; Q_1_, 1st quartile; Q_3_, 3rd quartile.

t, Student’s t test; t’, Satterthwaite t test; Z, Mann–Whitney U test; χ², Chi-square test; -, Fisher’s exact test.

The characteristics of patients included in the training group are presented in **Table 2**. This group included 180 male patients (54.88%) and 148 female patients (45.12%). Regarding medical history, 10 patients (3.05%) had a family history of cancer, 7 (2.13%) had a history of blood disorders, 54 (16.46%) suffered from hypertension, 26 (7.93%) had diabetes, 18 (5.49%) were diagnosed with coronary heart disease, 24 (7.32%) had cerebrovascular disease, and 6 (1.83%) were affected by autoimmune disease.

**Table 2:** Clinical characteristics of acute leukemia patients in the training set who experienced hemorrhage of WHO grade 2 or higher and those who did not.

Variables	Total (N=328)	WHO grade 2 or higher bleeding		Statistics	*P*
		No (n=129)	Yes (n=199)		
Demographic data					
Age, years, mean±SD	52.58±14.86	54.92±14.77	51.06±14.75	t=2.318	0.021
Sex, n (%)				χ²=4.374	0.036
Male	180 (54.88)	80 (62.02)	100 (50.25)		
Female	148 (45.12)	49 (37.98)	99 (49.75)		
Height, cm, mean±SD	165.69±7.85	166.37±7.83	165.25±7.86	t=1.264	0.207
Weight, kg, mean±SD	65.49±11.61	66.79±11.96	64.65±11.32	t=1.641	0.102
BMI, kg/m^2^, mean±SD	23.79±3.53	24.05±3.73	23.62±3.40	t=1.097	0.273
History of cancer, n (%)				χ²=0.081	0.776
No	318 (96.95)	125 (96.90)	193 (96.98)		
Yes	10 (3.05)	4 (3.10)	6 (3.02)		
History of hematological disease, n (%)				χ²=0.341	0.559
No	321 (97.87)	125 (96.90)	196 (98.49)		
Yes	7 (2.13)	4 (3.10)	3 (1.51)		
Smoking, n (%)				–	0.263
Never	266 (81.10)	102 (79.07)	164 (82.41)		
Former	9 (2.74)	6 (4.65)	3 (1.51)		
Current	53 (16.16)	21 (16.28)	32 (16.08)		
Drinking, n (%)				–	1
Never	299 (91.16)	117 (90.70)	182 (91.46)		
Former	2 (0.61)	1 (0.78)	1 (0.50)		
Current	27 (8.23)	11 (8.53)	16 (8.04)		
Hypertension, n (%)				χ²=0.709	0.4
No	274 (83.54)	105 (81.40)	169 (84.92)		
Yes	54 (16.46)	24 (18.60)	30 (15.08)		
Diabetes, n (%)				χ²=0.009	0.925
No	302 (92.07)	119 (92.25)	183 (91.96)		
Yes	26 (7.93)	10 (7.75)	16 (8.04)		
Coronary heart disease, n (%)				χ²=0.002	0.969
No	310 (94.51)	122 (94.57)	188 (94.47)		
Yes	18 (5.49)	7 (5.43)	11 (5.53)		
Cerebrovascular disease, n (%)				χ²=2.389	0.122
No	304 (92.68)	116 (89.92)	188 (94.47)		
Yes	24 (7.32)	13 (10.08)	11 (5.53)		
Autoimmune disease, n (%)				χ²=0.526	0.468
No	322 (98.17)	128 (99.22)	194 (97.49)		
Yes	6 (1.83)	1 (0.78)	5 (2.51)		
Comorbidities, n (%)				χ²=0.04	0.841
No	241 (73.48)	94 (72.87)	147 (73.87)		
Yes	87 (26.52)	35 (27.13)	52 (26.13)		
Disease features					
Anemia, n (%)				χ²=0.264	0.608
No	42 (12.80)	15 (11.63)	27 (13.57)		
Yes	286 (87.20)	114 (88.37)	172 (86.43)		
Extramedullary infiltration, n (%)				χ²=1.456	0.228
No	303 (92.38)	122 (94.57)	181 (90.95)		
Yes	25 (7.62)	7 (5.43)	18 (9.05)		
Prior bleeding, n (%)				χ²=6.061	0.014
No	254 (77.44)	109 (84.50)	145 (72.86)		
Yes	74 (22.56)	20 (15.50)	54 (27.14)		
Infection, n (%)				χ²=19.091	<0.001
No	142 (43.29)	75 (58.14)	67 (33.67)		
Yes	186 (56.71)	54 (41.86)	132 (66.33)		
Bone marrow cytomorphology					
Acute leukemia, n (%)				–	0.103
ALL	68 (20.73)	25 (19.38)	43 (21.61)		
AML	241 (73.48)	92 (71.32)	149 (74.87)		
Others	19 (5.79)	12 (9.30)	7 (3.52)		
Treatment information					
Treatment modality, n (%)				–	0.002
Chemotherapy	61 (18.60)	32 (24.81)	29 (14.57)		
Targeted therapy	258 (78.66)	90 (69.77)	168 (84.42)		
Both	9 (2.74)	7 (5.43)	2 (1.01)		
Types of hemorrhage prevention drugs and blood products, n (%)				–	<0.001
No	26 (7.93)	20 (15.50)	6 (3.02)		
1	262 (79.88)	97 (75.19)	165 (82.91)		
≥2	40 (12.20)	12 (9.30)	28 (14.07)		
Platelet transfusion, n (%)				χ²=17.44	<0.001
No	31 (9.45)	23 (17.83)	8 (4.02)		
Yes	297 (90.55)	106 (82.17)	191 (95.98)		
Treatment courses, times, M (Q_1_, Q_3_)	1.00 (1.00, 3.25)	1.00 (1.00, 4.00)	1.00 (1.00, 3.00)	Z=-1.272	0.203
Laboratory tests (post-treatment)					
WBC count, 10^9^/L, M (Q_1_, Q_3_)	1.98 (0.71, 5.01)	3.32 (1.53, 6.26)	1.10 (0.51, 4.16)	Z=-5.102	<0.001
NEUT count, 10^9^/L, M (Q_1_, Q_3_)	0.58 (0.12, 2.46)	1.50 (0.33, 3.41)	0.33 (0.07, 1.61)	Z=-5.447	<0.001
LYM count, 10^9^/L, M (Q_1_, Q_3_)	0.72 (0.41, 1.37)	0.87 (0.53, 1.57)	0.61 (0.34, 1.17)	Z=-3.497	<0.001
RBC count, 10^12^/L, mean±SD	2.44±0.69	2.55±0.73)	2.38±0.65)	t=2.199	0.029
Hb, g/L, mean±SD	75.10±21.04	77.96±22.41)	73.24±19.95)	t=1.993	0.047
HCT, %, mean±SD	23.00±6.53	24.10±6.92)	22.28±6.18)	t=2.474	0.014
PLT count, 10^9^/L, M (Q_1_, Q_3_)	27.00 (11.00, 70.00)	49.00 (21.00, 119.00)	18.00 (8.00, 41.00)	Z=-5.61	<0.001
FBG, g/L, mean±SD	3.80±2.14	3.62±1.88	3.92±2.29	t’=-1.268	0.206
PT, s, mean±SD	13.26±4.32	12.68±3.41	13.63±4.79	t=-1.961	0.051
APTT, s, mean±SD	29.72±6.36	29.59±6.42	29.81±6.33	t=-0.312	0.756
TT, s, mean±SD	15.14±3.50	15.15±3.15	15.13±3.72	t=0.045	0.964
TBIL, μmol/L, M (Q_1_, Q_3_)	9.30 (6.90, 13.72)	9.80 (6.80, 14.00)	9.10 (6.95, 13.40)	Z=-0.494	0.622
ALT, U/L, M (Q_1_, Q_3_)	17.00 (11.00, 29.00)	16.00 (11.00, 28.00)	18.00 (12.00, 29.00)	Z=-0.637	0.524
AST, U/L, M (Q_1_, Q_3_)	17.00 (12.00, 27.00)	16.00 (12.00, 25.00)	18.00 (12.40, 29.50)	Z=-1.117	0.264
GGT, U/L, M (Q_1_, Q_3_)	35.00 (21.00, 66.25)	31.00 (20.00, 59.00)	37.00 (22.50, 69.50)	Z=-1.04	0.299
ALP, U/L, M (Q_1_, Q_3_)	71.35 (55.68, 100.15)	75.20 (57.00, 101.00)	69.50 (54.60, 97.70)	Z=-1.255	0.21
BUN, mmol/L, M (Q_1_, Q_3_)	5.13 (3.79, 6.29)	5.12 (3.79, 6.04)	5.15 (3.74, 6.49)	Z=-0.536	0.592
Cr, μmol/L, M (Q_1_, Q_3_)	60.85 (50.98, 75.01)	62.10 (52.00, 73.80)	59.50 (49.30, 76.20)	Z=-0.696	0.486
Outcome					
Bleeding grade, n (%)				–	<0.001
No	91 (27.74)	91 (70.54)	0 (0.00)		
Grade 1	38 (11.59)	38 (29.46)	0 (0.00)		
Grade 2	164 (50.00)	0 (0.00)	164 (82.41)		
Grade 3	28 (8.54)	0 (0.00)	28 (14.07)		
Grade 4	7 (2.13)	0 (0.00)	7 (3.52)		
Treatment efficacy, n (%)				–	<0.001
Complete remission	121 (36.89)	31 (24.03)	90 (45.23)		
Complete remission with incomplete hematologic recovery	5 (1.52)	2 (1.55)	3 (1.51)		
Partial remission	11 (3.35)	4 (3.10)	7 (3.52)		
No remission	94 (28.66)	41 (31.78)	53 (26.63)		
Bone marrow not assessed	97 (29.57)	51 (39.53)	46 (23.12)		
Mortality, n (%)				χ²=7.521	0.006
No	251 (76.52)	109 (84.50)	142 (71.36)		
Yes	77 (23.48)	20 (15.50)	57 (28.64)		
Recurrence, n (%)				χ²=17.081	<0.001
No	218 (66.46)	103 (79.84)	115 (57.79)		
Yes	110 (33.54)	26 (20.16)	84 (42.21)		
Progression, n (%)				χ²=0.269	0.604
No	317 (96.65)	126 (97.67)	191 (95.98)		
Yes	11 (3.35)	3 (2.33)	8 (4.02)		
EFS, days, M (Q_1_, Q_3_)	152.50 (39.00, 364.25)	63.00 (24.00, 228.00)	214.00 (84.00, 475.50)	Z=-6.093	<0.001
OS, days, M (Q_1_, Q_3_)	196.50 (39.75, 432.25)	67.00 (24.00, 271.00)	256.00 (101.50, 518.00)	Z=-6.302	<0.001

SD, standard deviation; M, median; Q_1_, 1st quartile; Q_3_, 3rd quartile.

t: Student’s t test; t’: Satterthwaite t test; Z: Mann–Whitney U test; χ²: Chi-square test; -: Fisher’s exact test.

Within the training group, 199 patients with acute leukemia experienced hemorrhagic events related to anti-tumor therapy and of WHO grade 2 or higher. The distribution of the grades of these events for patients in the training group was as follows: 91 (27.74%) non-bleeding events, 38 (11.59%) grade 1 events, 164 (50.00%) grade 2 events, 28 (8.54%) grade 3 events, and 7 (2.13%) grade 4 events.

### Construction of predictive nomogram

**Figure 1:**
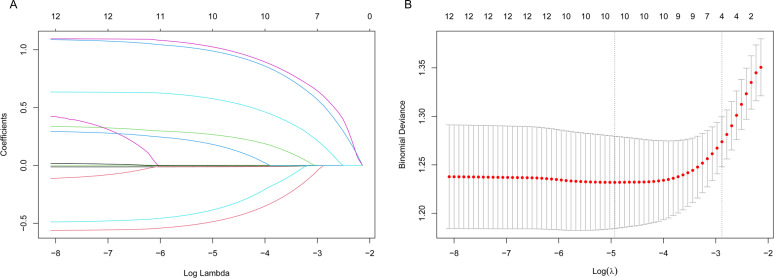
**(A)** Predictor identification using univariable logistic analysis; and **(B)** predictor selection based on LASSO regression. Instructions for Using the Nomogram: To estimate an individual patient’s risk of hemorrhage, a clinician should: For each of the five predictors, locate the patient’s value on the corresponding axis. Draw a vertical line straight down to the “Points” axis at the top to determine the score for that variable. Sum the points for all five variables to obtain a “Total Points” score. Locate this total on the “Total Points” axis and draw a vertical line straight down to the “Risk of Hemorrhage” axis at the bottom to read the predicted probability.

**Figure 2:**
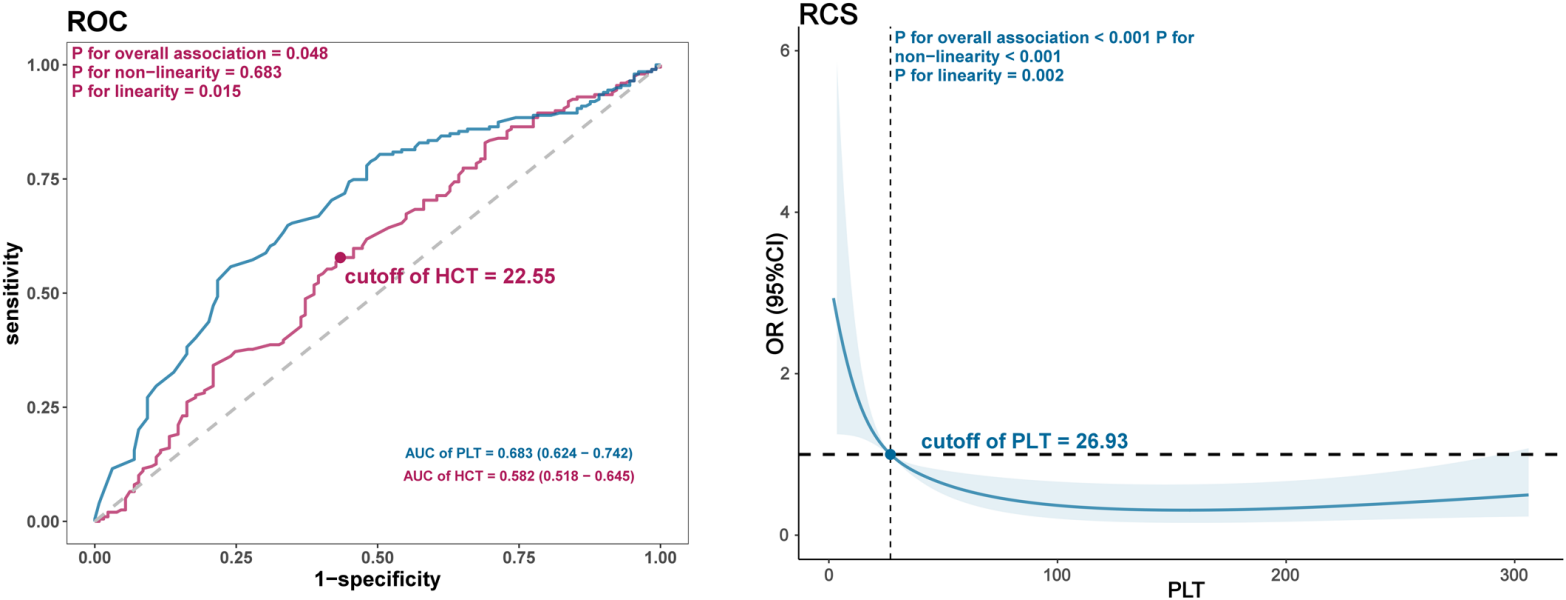
ROC curve with Youden index and RCS curve for calculation of the optimal cutoff values for HCT and PLT count, respectively.

**Figure 3:**
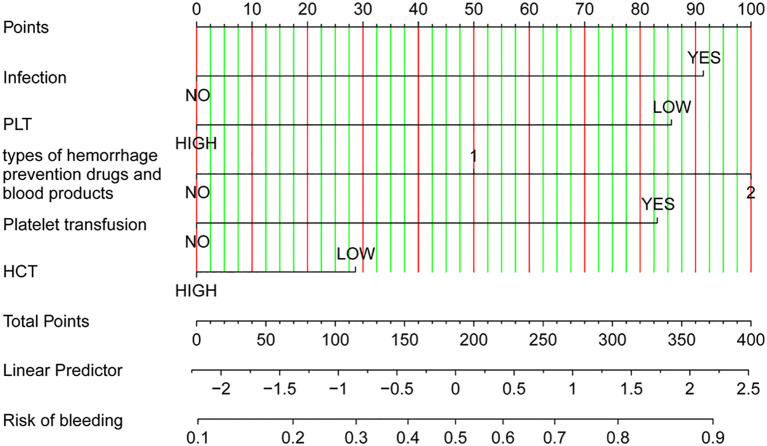
Nomogram for predicting the risk of anti-tumor therapy-related hemorrhage of WHO grade 2 or higher among acute leukemia patients.

Univariable logistic analysis of the training group data revealed that age, sex, infection, types of different hemorrhage prevention drugs and blood products administered, PLT transfusion, RBC count, Hb, treatment modality, HCT, and PLT count, prior bleeding were significantly associated with the occurrence of hemorrhage (*P*<0.05; **Table 3**). Further LASSO regression analysis and multivariable logistic analysis demonstrated that only infection, types of different hemorrhage prevention drugs and blood products administered, PLT transfusion, HCT, and PLT count were significant factors suitable for use in constructing the predictive model (**Tables 4** and **5** and **Figure 1**). The model was subjected to collinearity and goodness-of-fit testing, which demonstrated the consistency and excellence of the model fit with no evidence of collinearity among the variables (**Figure 1**). Because HCT followed a linear distribution, the optimal cutoff value was determined using the ROC curve and Youden index. For PLT count, which exhibited a nonlinear distribution, the restricted cubic spline (RCS) method was employed. The optimal cutoff values for HCT and PCT count were determined to be 22.55% and 26.93×10^9^/L, respectively (**Figure 2**). Subsequently, a nomogram was constructed to provide a visual representation of the prediction model (**Figure 3**).

**Table 3:** Univariate analysis of clinical characteristics of acute leukemia patients associated with the risk of WHO grade 2 or higher hemorrhage.

Variables	B	Wald	OR (95%CI)	*P*
Age	-0.018	5.237	0.982 (0.967-0.997)	0.022
Sex	-0.480	4.349	0.619 (0.394-0.972)	0.037
BMI	-0.035	1.201	0.966 (0.907-1.028)	0.273
History of cancer	-0.029	0.002	0.972 (0.269-3.512)	0.965
History of hematological disease	-0.737	0.912	0.478 (0.105-2.173)	0.340
Smoking		2.648		0.266
Never			Ref	
Former	-1.168	2.645	0.311 (0.076-1.271)	0.104
Current	-0.054	0.030	0.948 (0.518-1.733)	0.862
Drinking		0.121		0.941
Never			Ref	
Former	-0.442	0.097	0.643 (0.04-10.379)	0.756
Current	-0.067	0.027	0.935 (0.419-2.085)	0.870
Hypertension	-0.253	0.707	0.777 (0.431-1.4)	0.401
Diabetes	0.04	0.009	1.04 (0.457-2.37)	0.925
Coronary heart disease	0.02	0.002	1.02 (0.385-2.703)	0.969
Cerebrovascular disease	-0.65	2.324	0.522 (0.226-1.204)	0.127
Autoimmune disease	1.194	1.175	3.299 (0.381-28.567)	0.278
Comorbidities	-0.051	0.04	0.95 (0.576-1.567)	0.841
Anemia	-0.176	0.263	0.838 (0.427-1.645)	0.608
Extramedullary infiltration	0.55	1.426	1.733 (0.703-4.275)	0.232
Prior bleeding	0.708	5.924	2.03 (1.148-3.589)	0.015
Infection	1.007	18.642	2.736 (1.733-4.321)	<0.001
Acute leukemia		4.503		0.105
ALL			Ref	
AML	-0.06	0.045	0.942 (0.539-1.644)	0.832
Others	-1.081	4.04	0.339 (0.118-0.974)	0.044
WBC count	-0.004	1.091	0.996 (0.99-1.003)	0.296
NEUT count	-0.007	1.344	0.993 (0.981-1.005)	0.246
LYM count	-0.008	1.215	0.992 (0.979-1.006)	0.270
RBC count	-0.359	4.714	0.698 (0.505-0.966)	0.030
Hb	-0.011	3.896	0.989 (0.979-1)	0.048
HCT	-0.043	5.926	0.958 (0.926-0.992)	0.015
PLT count	-0.004	9.934	0.996 (0.993-0.998)	0.002
FBG	0.069	1.453	1.071 (0.958-1.198)	0.228
PT	0.063	3.483	1.065 (0.997-1.138)	0.062
APTT	0.006	0.098	1.006 (0.971-1.042)	0.755
TT	-0.001	0.002	0.999 (0.937-1.064)	0.964
TBIL	-0.002	0.156	0.998 (0.986-1.009)	0.693
ALT	0.001	0.164	1.001 (0.996-1.006)	0.686
AST	0.002	0.467	1.002 (0.996-1.008)	0.494
GGT	0	0.053	1 (0.997-1.002)	0.817
ALP	-0.002	0.78	0.998 (0.994-1.002)	0.377
BUN	0.016	0.183	1.017 (0.943-1.096)	0.668
Cr	0.002	0.184	1.002 (0.994-1.009)	0.668
Treatment modality		10.855		0.004
Chemotherapy			Ref	
Targeted therapy	0.723	6.306	2.06 (1.172-3.62)	0.012
Both	-1.154	1.88	0.315 (0.061-1.641)	0.170
Types of different hemorrhage prevention drugs and blood products		14.349		<0.001
No			Ref	
1	1.735	12.92	5.67 (2.201-14.605)	<0.001
≥2	2.051	12.534	7.778 (2.498-24.214)	<0.001
PLT transfusion	1.645	14.773	5.18 (2.239-11.985)	<0.001
Number of treatment courses	-0.063	2.589	0.939 (0.869-1.014)	0.108

OR, odds ratio; CI, confidence interval; Ref, reference.

**Table 4:** LASSO regression coefficients and collinearity test for factors associated with anti-tumor therapy-related hemorrhage in acute leukemia patients.

Variables	Beta	VIF
(Intercept)	-0.649799392	
Infection	0.516647335	1.024541
PLT transfusion	0.599423885	1.116979
Types of hemorrhage prevention drugs and blood products	0.168284131	1.126291
HCT	-0.009589530	1.215247
PLT count	-0.001333247	1.226876

VIF, variance inflation factor.

**Table 5:** Multivariate analysis of clinical factors associated with WHO grade 2 and above bleeding risk in acute leukemia patients after anti-tumor therapy.

Variables	B	Wald	OR (95% CI)	*P*
Infection	1.092	18.671	2.979 (1.816-4.888)	<0.001
HCT-binary	-0.536	4.954	0.585 (0.365-0.938)	0.026
PLT count-binary	-1.051	14.502	0.350 (0.204-0.601)	<0.001
Types of hemorrhage prevention drugs and blood products		11.250		0.004
No			Ref	
1	1.694	10.750	5.439 (1.976-14.967)	0.001
≥2	1.860	8.896	6.423 (1.892-21.806)	0.003
PLT transfusion	1.301	8.056	3.674 (1.496-9.022)	0.005

### Evaluation of predictive model performance

**Figure 4:**
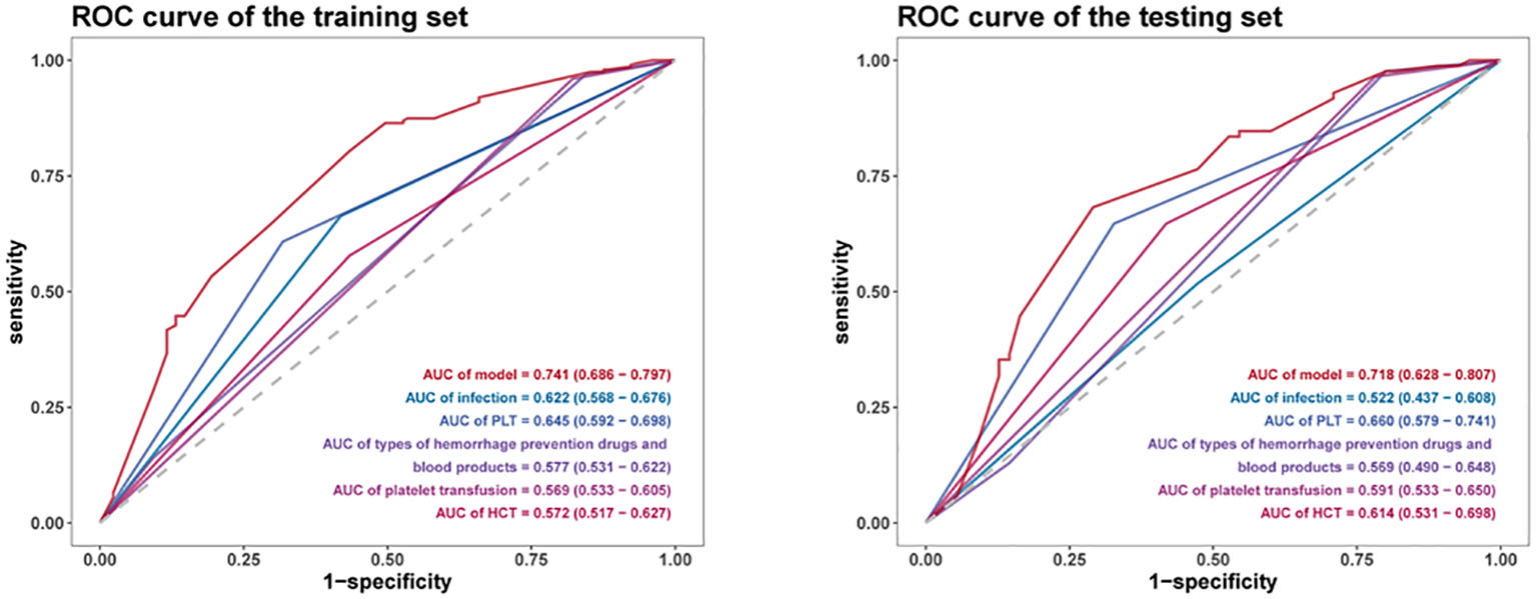
ROC curve analysis of the predictive performance of the developed nomogram in the training and test groups.

**Figure 5:**
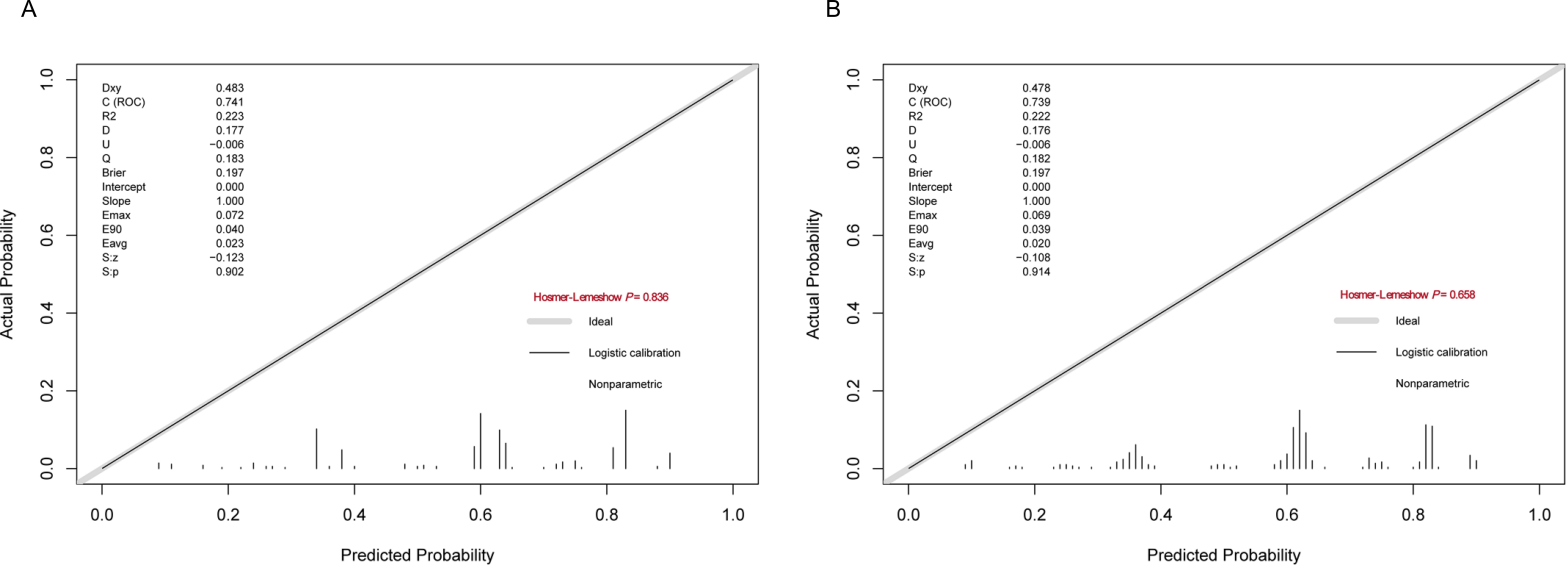
Calibration curves for the performance of the developed nomogram in the **(A)** training group and **(B)** test group.

**Figure 6:**
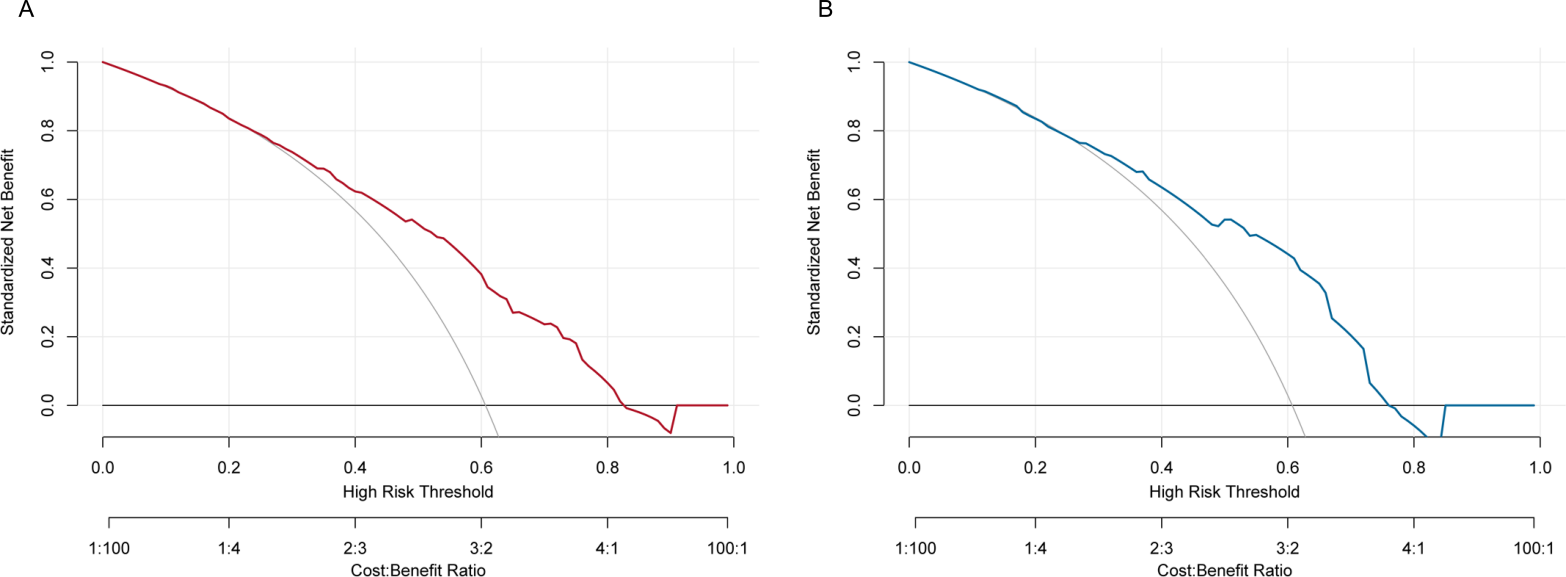
DCA of the clinical value of the developed nomogram in the **(A)** training group and **(B)** test group.

The predictive performance of the developed nomogram was validated in both the training group and test group by ROC curve analysis, calibration curve analysis, and DCA. As shown in **Figure 4**, the model demonstrated satisfactory performance discriminatory capacity, with the ROC curve yielding area under the curve (AUC) values of 0.741 (95% CI: 0.636–0.797) and 0.718 (95% CI: 0.628–0.807) in the training and test sets, respectively. These results indicate that the nomogram is effective at predicting the risk of anti-tumor therapy-related hemorrhage of WHO grade 2 or higher in acute leukemia patients. Additionally, the calibration plots showed excellent consistency between the actual and nomogram-predicted survival rates in the training group (**Figure 5A**) and test group (**Figure 5B**). Furthermore, DCA demonstrated that the nomogram had a high net benefit, indicating its clinical application value (**Figure 6**).

**Figure 7:**
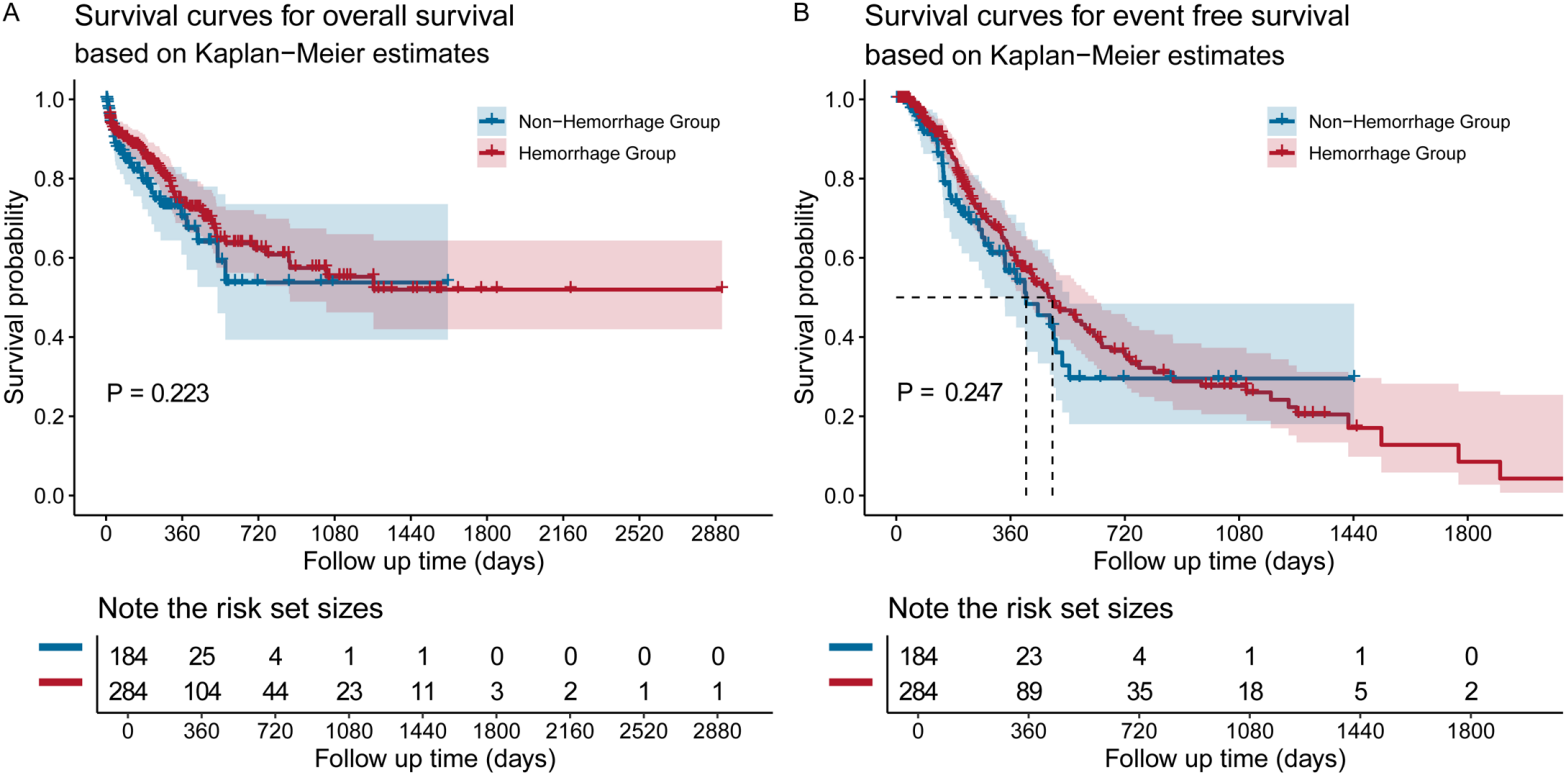
Kaplan–Meier survival analysis for **(A)** OS and **(B)** EFS among acute leukemia patients who experienced WHO grade 2 or higher hemorrhagic events and those who did not.

Finally, Kaplan–Meier survival analysis showed no significant differences in OS (**Figure 7A**) and EFS (**Figure 7B**) between acute leukemia patients who experienced WHO grade 2 or higher hemorrhagic events and those who did not.

## Discussion

The present study investigated potential associations of clinicopathological characteristics of acute leukemia patients with the risk of hemorrhage related to anti-tumor therapies. Univariable logistic analysis and LASSO regression analyses identified five factors that were significantly associated with the risk of anti-tumor therapy-related hemorrhage of WHO grade 2 or higher. Using these factors, we developed a novel nomogram for predicting hemorrhage risk in patients with acute leukemia. Based on the results of ROC curve analysis, calibration curve analysis, and DCA, the developed nomogram demonstrated good accuracy and clinical utility.

Chemotherapy involving cytotoxic drugs or the combination of chemotherapy with targeted therapy remains the main therapeutic strategy for patients with acute leukemia (18, 19). Chemotherapy-induced thrombocytopenia (CIT), a common complication of cytotoxic drug chemotherapy and many targeted therapies, can result in delayed chemotherapy, dosage reductions, and treatment interruptions, and puts patients at risk for hemorrhagic complications (20). In the present study, five clinical factors, including infection, types of different hemorrhage prevention drugs and blood products administered, PLT transfusion, HCT, and PLT count, were found to be linked to the risk of anti-tumor therapy-related hemorrhage of WHO grade 2 or higher. Hemorrhage is often associated with coagulation dysfunction, with symptom severity correlating with the degree of coagulation issues (21). However, in this study, the identified risk factors did not include the prothrombin time (PT) or activated partial thromboplastin time (APTT), main indicators of coagulation function. In a previous study, coagulation dysfunction at the level of DIC was found to be the key driver of WHO grade 4 (fatal or disabling) hemorrhagic events, while no significant link was found between coagulation abnormalities and grade 2–3 bleeding, indicating that milder events have more heterogeneous clinical presentations and are likely influenced by multiple mechanisms (22). Furthermore, in the present study, sex was a significant factor on univariable analysis but was subsequently excluded in the LASSO analysis, indicating that sex is not an independent risk factor in this population. Our results also revealed that certain factors previously linked to bleeding risk (such as age, Hb level, liver/kidney function, and WBC count) were not independently associated with WHO grade 2 or higher hemorrhage in our study population. A previous study by Xu et al. on AML patients identified infection and respiratory failure as factors independently related to coagulation (23). The types of different hemorrhage prevention drugs and blood products administered were also independently associated with the risk of anti-tumor therapy-related hemorrhage of WHO grade 2 or higher.

In the present study, if a patient had a PLT count of ≤100×10^9^/L, one or more of the following hemorrhage prevention drugs and blood products might be administered: thrombopoietin (TPO), serum interleukin-11 (IL-11), prothrombin complex, plasma, cryoprecipitate, FBG, recombinant activated factor VII, etamsylate, and tranexamic acid. Previous research has shown that PLT transfusion and PLT count are related to the risk of intracranial hemorrhage among acute leukemia patients, and even after adjustment for PLT count, the link between PLT transfusion and intracranial hemorrhage remained. This risk usually remained stable or increased in cases with one or more PLT counts ≤10×10^9^/L and the high percentage of hours with a PLT count ≤20×10^9^/L (24). Consistent with this prior research, our results indicate that PLT transfusion and PLT count serve as significant and independent predictors for the risk of hemorrhagic events of WHO grade 2 or higher during anti-tumor therapy. This seemingly contradictory phenomenon warrants further investigation. In addition, HCT might affect PLT properties and thus influence the hemorrhage risk for patients with leukemia (25). RBCs can increase blood viscosity by raising HCT and increasing flow resistance. Conversely, anemia, associated with low blood viscosity, may lead to a hemorrhagic tendency because of reduced PLT margination toward endothelium and enhanced nitric oxide availability, which inhibits PLTs and dilates blood vessels [30,31].

Nomograms, as simple visual prediction tools, are useful for evaluating the probabilities of clinical outcomes in specific populations (26). Presently, nomograms are extensively utilized for predicting prognosis among cancer patients (27, 28). The present study established a nomogram for predicting the risk of anti-tumor therapy-related hemorrhage of WHO grade 2 or higher among acute leukemia patients. We validated the predictive power of this nomogram by ROC curve analysis and calibration curve analysis. In addition, DCA demonstrated the clinical benefit and utility of the developed nomogram for predicting the risk of hemorrhage. Consequently, this study provides new insights into the prediction of WHO grade 2 or higher hemorrhage related to anti-tumor therapy among patients with acute leukemia.

A notable finding of our study was that the occurrence of WHO grade ≥2 hemorrhagic events was not associated with significant differences in OS or EFS. This finding, which may seem counterintuitive, is likely multifactorial. The majority of bleeding events in our cohort were of moderate severity (Grade 2, 50%), with life-threatening (Grade 4) events being rare (~2%). Furthermore, the extensive use of prophylactic and therapeutic interventions, including PLT transfusions and hemostatic agents, in more than 90% of patients suggests that modern supportive care was effective at mitigating the independent impact of bleeding on mortality. Consequently, in our population, a hemorrhagic event may have served more as a marker of the underlying myelosuppressive state rather than as a direct, independent driver of poor prognosis. This contrasts with some historical reports and may reflect advances in contemporary transfusion and supportive care practices that have successfully attenuated the fatal consequences of treatment-related hemorrhage.

To the best of our knowledge, the present study is the first to investigate a method for predicting the risk of hemorrhage related to anti-tumor therapy among patients with acute leukemia based on the integration of clinical features. The developed nomogram holds promise as a valuable tool for predicting hemorrhage risk in this patient population. Nevertheless, this study also has some limitations. Firstly, the sample size was relatively limited. Secondly, the study did not include external validation of the developed nomogram, which is crucial for ensuring its robustness. Such validation is needed to enhance the credibility of our findings. Lastly, the prognosis of acute leukemia patients can vary widely based on different immune phenotypes, cell types, and molecular genetics, but this study did not include stratified analyses of prognosis in these patients. Further multi-center studies with large sample sizes are required to validate the clinical utility of the established prediction nomogram.

## Conclusion

This study identified infection, the types of different hemorrhage prevention drugs and blood products administered, PLT transfusion, HCT, and PLT count as independent predictors of the risk of anti-tumor therapy-related hemorrhage of WHO grade 2 or higher among patients with acute leukemia. The developed predictive nomogram based on these indicators demonstrated strong predictive ability and user-friendliness for clinicians. However, external validation of the nomogram is needed in future studies to confirm the reliability of the nomogram.

## Data Availability

The original contributions presented in the study are included in the article/**Supplementary Material**. Further inquiries can be directed to the corresponding author/s.
