# Supplementary material for: Nomogram to predict hemorrhage risk related to anti-tumor therapy in patients with acute leukemia
Source: Front Oncol. 2026 Feb 26;15:1684145. doi: 10.3389/fonc.2025.1684145 (PMC12979094; doi:10.3389/fonc.2025.1684145)
Supplement: Supplementary file 1 [file DataSheet1.docx]

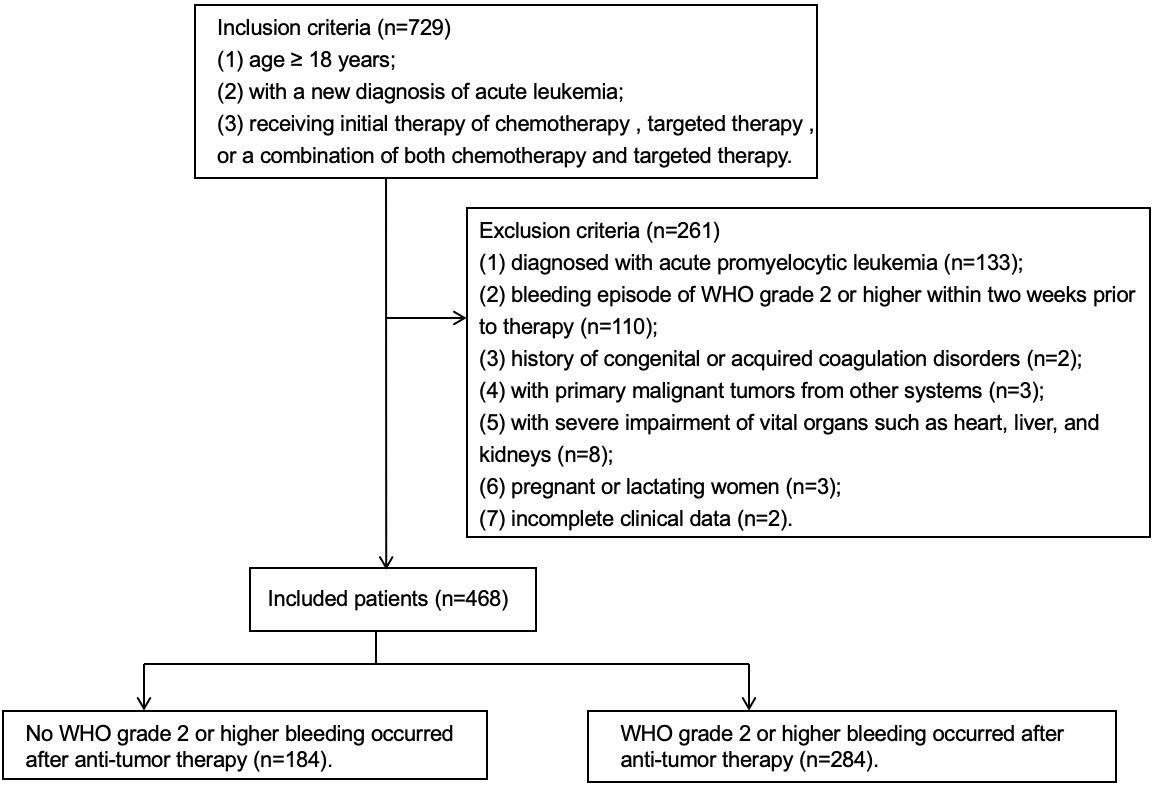


Supplemental Figure 1. Flow chart of patient selection.

Supplemental table 1, Clinical characteristics recorded for included patients and the corresponding rates of missing data.

| Variables | Missing values, n (%) |
| --- | --- |
| Height | 1 (0.00) |
| Neutrophil (NEUT) count | 25 (0.05) |
| Lymphocyte (LYM) count | 26 (0.06) |
| Platelet (PLT) count | 1 (0.00) |
| PLT distribution width | 105 (0.22) |
| Mean PLT volume | 106 (0.23) |
| PLT-larger cell ratio | 117 (0.25) |
| Fibrinogen (FBG) | 82 (0.18) |
| Prothrombin time (PT) | 81 (0.17) |
| Activated partial thromboplastin time (APTT) | 86 (0.18) |
| Thrombin time (TT) | 81 (0.17) |
| D-Dimer | 356 (0.76) |
| Total bilirubin (TBIL) | 13 (0.03) |
| Alanine aminotransferase (ALT) | 12 (0.03) |
| Aspartate aminotransferase (AST) | 14 (0.03) |
| Gamma-glutamyl transferase (GGT) | 25 (0.05) |
| Alkaline phosphatase (ALP) | 29 (0.06) |
| Blood urea nitrogen (BUN) | 11 (0.02) |
| Creatinine (Cr) | 12 (0.03) |
| Uric acid (UA) | 102 (0.22) |
| Treatment courses | 3 (0.01) |

Supplemental table 2, Sensitivity analysis before and after interpolation

| Variables | Before (N=468) | After (N=468) | Statistics | *P* |
| --- | --- | --- | --- | --- |
| Height, cm, mean ± SD | 165.47±7.97 | 165.46±7.96 | t=0.022 | 0.982 |
| NEUT count, 10^9^/L, M(Q₁, Q₃) | 0.58 (0.12, 2.25) | 0.62 (0.14, 2.36) | Z=-0.562 | 0.574 |
| LYM count, 10^9^/L, M(Q₁, Q₃) | 0.72 (0.38, 1.38) | 0.73 (0.39, 1.4) | Z=-0.292 | 0.770 |
| PLT count, 10^9^/L, M(Q₁, Q₃) | 26 (11, 67) | 26 (11, 66.75) | Z=-0.035 | 0.972 |
| FBG, g/L, mean ± SD | 3.74±2.01 | 3.74±2.04 | t=-0.049 | 0.961 |
| PT, s, mean ± SD | 13.33±4.15 | 13.27±4.18 | t=0.188 | 0.851 |
| APTT, s, mean ± SD | 29.77±6.59 | 29.70±6.44 | t=0.165 | 0.869 |
| TT, s, mean ± SD | 15.16±3.34 | 15.15±3.34 | t=0.043 | 0.966 |
| TBIL, μmol/L, M(Q₁, Q₃) | 9.3 (6.9, 13.6) | 9.3 (6.9, 13.6) | Z=-0.135 | 0.893 |
| ALT, U/L, M(Q₁, Q₃) | 17 (11, 29) | 16.5 (11, 29) | Z=-0.101 | 0.919 |
| AST, U/L, M(Q₁, Q₃) | 17 (12, 26) | 17 (12, 27) | Z=-0.013 | 0.990 |
| GGT, U/L, M(Q₁, Q₃) | 34 (20, 63.5) | 34 (20, 62.75) | Z=-0.034 | 0.973 |
| ALP, U/L, M(Q₁, Q₃) | 71.9 (56.2, 97.3) | 71 (56.1, 97.23) | Z=-0.318 | 0.751 |
| BUN, mmol/L, M(Q₁, Q₃) | 5.09 (3.74, 6.3) | 5.09 (3.73, 6.28) | Z=-0.123 | 0.902 |
| Cr, μmol/L, M(Q₁, Q₃) | 60.46 (50, 73.97) | 60.66 (50, 74.68) | Z=-0.107 | 0.915 |
| Treatment courses, times, M(Q₁, Q₃) | 1 (1, 3) | 1 (1, 3) | Z=-0.014 | 0.989 |

Note: SD: standard deviation; M: median; Q₁: 1st quartile; Q₃: 3rd quartile

t: Student’s t test; t': Satterthwaite t test; Z: Mann–Whitney U test
